# Preparation of Chitosan/Recombinant Human Collagen-Based Photo-Responsive Bioinks for 3D Bioprinting

**DOI:** 10.3390/gels8050314

**Published:** 2022-05-19

**Authors:** Yang Yang, Zixun Wang, Yuanyuan Xu, Jingjing Xia, Zhaoxian Xu, Shuai Zhu, Mingjie Jin

**Affiliations:** 1 Department of Mechanical Engineering, Tsinghua University, Beijing 100084, China; xu-yy18@mails.tsinghua.edu.cn (Y.X.); xiajj19@mails.tsinghua.edu.cn (J.X.); 2 School of Environmental and Biological Engineering, Nanjing University of Science and Technology, Nanjing 210094, China; wangzixun@njust.edu.cn (Z.W.); xzx018@njust.edu.cn (Z.X.); zhushuai@njust.edu.cn (S.Z.); jinmingjie@njust.edu.cn (M.J.)

**Keywords:** chitosan, recombinant human collagen, bioinks, 3D printing, tissue engineering

## Abstract

Collagen and chitosan are frequently used natural biomaterials in tissue engineering. However, most collagen is derived from animal tissue, with inconsistent quality and pathogen transmittance risks. In this context, we aimed to use a reliable Type-III recombinant human collagen (RHC) as an alternative biomaterial together with chitosan to develop novel photo-responsive bioinks for three-dimensional (3D) bioprinting. RHC was modified with methacrylic anhydride to obtain the RHC methacryloyl (RHCMA) and mixed with acidified chitosan (CS) to form composites CS-RHCMA. The characterizations demonstrated that the mechanical properties and the degradation of the bioinks were tunable by introducing the CS. The printabilities improved by adding CS to RHCMA, and various structures were constructed via extrusion-based 3D printing successfully. Moreover, in vitro tests confirmed that these CS-RHCMA bioinks were biocompatible as human umbilical vein endothelial cells (HUVECs) were sustained within the constructs post-printing. The results from the current study illustrated a well-established bioinks system with the potential to construct different tissues through 3D bioprinting.

## 1. Introduction

3D printing with cell-laden bioinks is an advanced technology based on additive manufacturing [1,2,3] applied to the biomedical field and tissue engineering [4,5,6]. Compared to 2D culture conditions, bioprinted 3D constructs mimic the biological microenvironment of native tissue, therefore, supporting regular cell activities [7,8,9]. Bioinks development is one of the key elements to successful 3D bioprinting [10,11]. Meanwhile, the tissue specificity of the bioink promotes cell adhesion and differentiation, thus assisting the formation of functional tissues. For instance, adding collagen or other biological components to the ink can represent the extracellular matrix (ECM), leading to a stable cell niche [12,13]. Various crosslinking mechanisms, such as chemistry [14], light [15], and heat [16], are used to crosslink the printed bioinks resulting in gelled constructs to mimic the specific native tissues, such as skin [17], cartilage, or bone [18,19,20].

Recently, biomaterials have received great attention, and there has been major progress in their design and synthesis to provide them with the biological and mechanical properties of the native tissue. The latest progress includes the development of bioinks from advanced biomaterials and their use in 3D bioprinting. Designing advanced bioinks always relies on the synthesis of polymers or modification of natural polymers. Collagen [21], gelatin [22], hyaluronic acid [23], sodium alginate [24], chitosan [25], and cellulose [26] are biomaterials obtained from natural products frequently used in preparing bioinks.

Collagen [27] and chitosan [28] are excellent biomaterials. They have been widely used in engineering tissue scaffolds and regenerative medicine, including wound dressing, drug carriers, and tissue regeneration. Chitosan, the deacetylated derivative of chitin, is a linear polysaccharide composed of glucosamine and N-acetyl-glucosamine residues. Chitosan with high molecular weight can only be dissolved in acidic solutions via primary amine protonation; the lack of solubility at neutral pH hinders chitosan utilization in certain applications under physiological conditions, especially under their gelled forms [29]. Plenty of works have proved the feasibility of chitosan for tissue engineering scaffolds, wound dressings, and drug delivery applications due to its excellent biocompatibility, controllable degradation rate, tunable mechanical properties, and bacteriostatic effects [30,31,32]. Collagen belongs to the protein family, and it is known as a key structural protein of the ECM that supports the morphology and integrity of the native tissue. Therefore, it is widely applied in tissue engineering. However, commercial collagen products are mainly animal tissue-derived, and they are normally reserved in an acid solution. Moreover, a neutralization process is always required to sustain the cell viabilities once cells are loaded into collagen-based precursors [33]. Therefore, it hampers the efficiency of making the cell-loaded tissue engineering scaffolds, especially for the 3D bioprinting products. Other concerns of using animal-sourced collagen are batch-to-batch variations resulting in inconsistent reproducibility, immunogenicity, and the possibility of animal virus transmission [15,34]. Recombinant human collagen (RHC) could be an alternative to animal-sourced collagen to overcome the defects mentioned above. Recombinant human collagen is high in purity and highly reproducible from the fermentation of microorganisms or transgenic crops. These virus-free products share the same amino acid sequence as human collagen. In addition, some of the recombinant human collagen have a well-defined molecular structure with excellent solubility in aqueous conditions, advancing the formation of cell-loaded gels in 3D bioprinting [35]. Our team previously designed multiple tissue engineering scaffolds with self-constructed RHC and chitosan [36,37,38]; these works have fully verified that our RHC has an extensive application prospect in tissue engineering and have laid a good foundation for the development of chitosan-collagen bioinks in this research.

This study adopted a reliable, predictable, and chemically-defined Type-III recombinant collagen (RHC) developed in the Bioengineering Lab of Nanjing University of Science and Technology together with commercial chitosan (CS) to fabricate 3D printable bioinks. We aimed to explore the feasibility of using these CS-RHCMA as reliable bioinks in tissue engineering. These CS-RHCMA bioinks and their UV-cured constructs were then comprehensively investigated. Fourier transform infrared spectroscopy (FT-IR) was used to determine the success of producing the RHCMA through MAA modification. The internal morphology of the UV-cured bioinks was investigated using scanning electron microscopy (SEM) after freeze-drying. The mechanical strength, as well as the biodegradation of the UV-cured bioinks, was carefully determined. Lattices were printed by extrusion-based 3D printing to evaluate the printability of the bioinks, and the viscosity values of the bioinks were assessed. The cytotoxicity of the bioinks was assessed by the sample elution test according to ISO-10993:5. HUVECs were pre-loaded in CS-RHCMA bioinks and printed to form cell-laden constructs where cell survivability and morphology were determined. Our results indicated that the bioinks based on chitosan and RHC demonstrated tunable mechanical properties. These bioinks showed ideal printability compared to RHCMA only bioinks and had no toxicity to the cells. The 3D printed constructs also showed their capability to maintain the viability of HUVECs, thus laying the foundation for its application in the tissue engineering field in the future.

## 2. Materials and Methods

### 2.1. Materials

Type III recombinant human collagen (Mw. 112 kD, The National Center for Biotechnology Information GenBank Access Number: EF376007.1) with high hydrophilicity was prepared by our laboratory [39]. Methacryllc anhydride (MAA, 94%, containing 0.2% topanol stabilizer) was purchased from Aladdin (Shanghai, China). Chitosan (Mw. 100-300 KD) was purchased from J&K Scientific (Beijing, China). Lithium phenyl-2,4,6-trimethylbenzoyl phosphinate (LAP) was purchased from Macklin. The Cell Counting Kit-8 reagent kit (CCK-8) was purchased from Beyotime Biotechnology (Shanghai, China). The acridine orange/ethidium bromide (AO/EB) assay kit was purchased from Yeasen Biotech (Shanghai, China), and other chemical reagents for analysis on quality were purchased from Sinopharm Chemical Reagent (Shanghai, China). The cell culture medium and reagents were purchased from GIBCO (Shanghai, China). The HUVECs were purchased from ZhongQiaoXinZhou Biotechnology Co., Ltd. (Shanghai, China).

### 2.2. Preparation of the CS-RHCMA Bioinks

**Figure 1:**
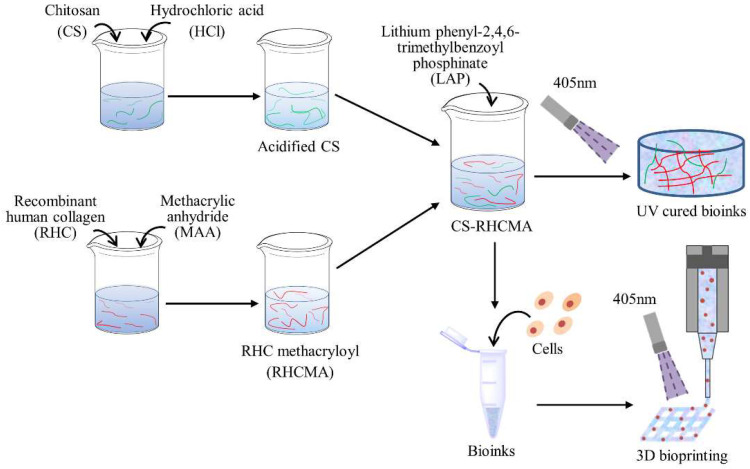
Schematic diagram of preparation of the CS-RHCMA bioinks.

A schematic diagram of the bioinks preparation is shown in Figure 1. To prepare the acidified chitosan, 2 g chitosan was weighed and dissolved in 100 mL 0.1M hydrochloric acid solution with magnetic stirring at 1000 r/min at 37 °C for 5 h to ensure the chitosan dissolved completely. Next, the chitosan solution was transferred into a dialysis bag (36 MM, MW: 14,000) and dialyzed for 5 d at 37 °C to remove the surplus hydrochloric acid from the chitosan solution. Then, the solution was freeze-dried (LGJ-10D, Beijing, China) to obtain the acidified CS powder.

To obtain the RHC methacryloyl, 4.8 mL MAA was added dropwise into 40 mL 15% (*w*/*v*) RHC phosphate solution, stirred for 1 h, and then centrifuged to collect the supernatant. Deionized water was added to dilute the supernatant, and dialysis was conducted for 4 d at 37 °C with a dialysis bag (36 MM, MW: 14,000) to filter the unreacted methacrylic anhydride. The pH value was adjusted to 7, and the solution was freeze-dried to collect the MAA-modified recombinant human collagen (RHCMA) powder.

The CS and RHCMA powder were dissolved in PBS solution to prepare the 1% (*w*/*v*) CS and 10% (*w*/*v*) RHCMA solution, respectively. The CS and the RHCMA solution were mixed at mass ratios of 1:3, 2:3, and 3:3, and final concentrations were set to 10% (*w*/*v*). The pH of the mixtures was carefully adjusted to 7 with 1 M sodium hydroxide. An amount of 0.25% (*w*/*v*) LAP was added to these mixtures; they were stirred in the dark for 1 h at room temperature, thus preparing three types of photo-responsive CS-RHCMA bioinks.

### 2.3. FT-IR Characterization

Infrared spectra of the CS, acidified CS, RHC, RHCMA, and CS-RHCMA were determined with a Nicolet IS-10 infrared microscope (Thermo Fisher Scientific, Waltham, MA, USA). All spectra were recorded in the absorption mode at 2 cm^−1^ intervals in the wavelength range of 4000–500 cm^−1^, where freeze-dried samples were tested.

### 2.4. Morphological Characterization

The scanning electron microscope (ZEISS Sigma500, Jena, Germany) was adopted to observe the internal morphology of UV-cured CS-RHCMA bioinks. Before the SEM observation, the freeze-dried samples were sectioned using a scalpel and sputter-coated with gold for 90 s. The average pore size was calculated by measuring the diameters of randomly selected pores.

### 2.5. Mechanical Testing

The mechanical properties of the UV-cured bioinks were evaluated via the stress-strain test with a universal mechanical test system (UTM6104, Shenzhen, China). The cylindrical samples with a diameter of 20 mm and a height of 5 mm were compressed at a constant speed of 10 mm/min up to 70% strain. The test instrument collected the force and deformation data and then converted them into stress-strain values. The compressive modulus was determined according to the slope of the linear portion of the 10% strain for all samples.

### 2.6. Biodegradation

For biodegradation tests, UV-cured bioinks samples were subjected to either PBS buffer (pH 7.2) or PBS buffer containing lysozyme (6.8 g/L, pH 6.5). Samples were weighed and then immersed in the test solution to determine their degradation behavior. The solution was exchanged every 2 days, and the incubation temperature was set to 37 °C. At each defined time point, samples were removed from the solution and rinsed with distilled water. Filter paper was used to remove excess water, and samples were weighed immediately. The weight changes that represented the biodegradation status were calculated as follows: (Wt − Wo)/Wo × 100%, where Wo is the initial weight of the UV-cured samples and Wt is the weight of the degraded samples at a specific time point [40].

### 2.7. Viscosity and Printability Test

The viscosity values associated with the rheological properties of the bioinks were determined by a rheometer (MCR302, Anton Paar, Austria) equipped with a 25 mm plate (CP-25), and tested with an angular frequency of 10 rad/s. The gap distance was 50 μm; all tests were conducted at room temperature. To determine the printability of the CS-RHCMA bioinks, grid structures were printed by an extrusion-based 3D bioprinter (Bio-X, CELLINK, Sweden) equipped with a 25G nozzle. The printing speed was 16 mm/s, and the extrusion volume was 0.07 μL/mm. A 405 nm UV (25 mW/cm^2^) light source was adopted to solidify the printed constructs. Printability is defined based on a square shape using the following equation quoted from a previous study: Printability = L^2^/16A, where L is the perimeter of the single grid, and A refers to the area within this grid [41]. Additionally, slices, lattices, and porous cubes were also printed to demonstrate the printability of CS-RHCMA.

### 2.8. Cell Culture

F-12K complete medium containing 10% fetal bovine serum (FBS) and antibiotics (100 IU/mL penicillin and 100 ug/mL streptomycin) was used as the culture medium for the HUVECs, and cells were cultured at 37 °C in a 5% CO_2_ humidified incubator.

### 2.9. Cytotoxicity Test

The cytotoxicity of the CS-RHCMA was evaluated via a modified elution test according to the ISO-10993:5. First, 0.5 mL bioinks were added into each well of a 12-well plate and then cured using UV irradiation. Next, each sample was taken out and immersed in 2.5 mL F-12K. The elution was collected after incubating for 24 h at 37 °C. Meanwhile, HUVECs were seeded in the 96-well plate with a density of 3000 cells/cm^2^, and cultured at 37 °C in a 5% CO_2_ humidified incubator. After one day of culture, the medium was replaced with extracted eluents. Cells cultured in the fresh medium were used as a control. An amount of 20 uL CCK-8 was added to each sample at defined culture times (24 h and 72 h) and incubated for another 3 h. A microplate reader (Infinite M200 PRO, Tecan, Vienna, Austria) was used to determine the absorbance at 450 nm. According to ISO 10993:5, the sample was considered cytotoxic if the cellular viability was reduced to less than 70% of the control group. The status of the HUVECs was examined by AO/EB double-fluorescent staining. The AO stained viable cells and exhibited a bright green intact structure, whereas the EB stained apoptotic cells and appeared as a red-orange color. Cellular morphology was observed under an inverted fluorescence microscope (X-81, Olympus, Shinjuku, Japan).

### 2.10. 3D Bioprinted Constructs of HUVECs-Laden CS-RHCMA Bioinks

HUVECs were collected firstly and mixed with CS-RHCMA to obtain bioinks with a cell density of 1 × 10^6^ cells/mL. Grid constructs with 500 µm thickness were then printed with the same parameters in the printability tests and UV-cured for 45 s. The solidified constructs were incubated in a fresh medium. AO/EB double-fluorescent staining was used to identify the viability of the HUVECs within the constructs at 1 h and 48 h post-printing (laser confocal microscopy-FV3000, Olympus). The survivability of the HUVECs post-printing was determined by ImagePro Plus from the captured fluorescent images. The ImagePro plus software was used to identify the green living cells and red dead cells.

### 2.11. Statistical Analysis

At least three samples of each group were tested. Data collected from each experiment are presented as mean ± standard deviation and plotted with OriginPro 2017 (Origin Laboratories, San Francisco, CA, USA). Differences were detected by the t-test; significance is indicated with * *p* < 0.05 and ** *p* < 0.01, where *p* > 0.05 was considered not statistically significant.

## 3. Results and Discussion

### 3.1. Characterization of the CS-RHCMA

**Figure 2:**
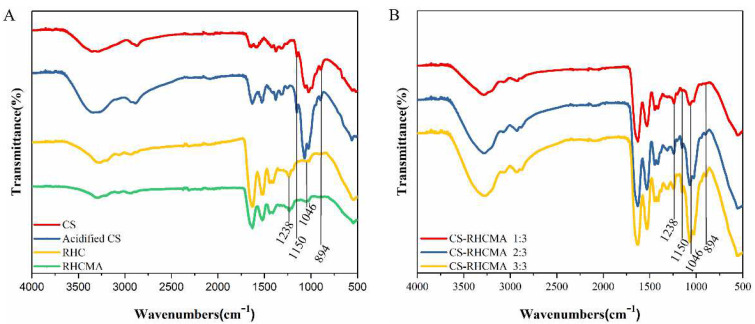
Characterization of functional groups of the prepared samples. (**A**) FT-IR spectra of CS, acidified CS, RHC, and RHCMA. (**B**) FT-IR spectra of CS-RHCMA with different CS to RHCMA ratios.

The molecule structures of CS, acidified CS, RHC, RHCMA, and CS-RHCMA were analyzed by FT-IR, and the results are shown in Figure 2. In CS (Figure 2A), the typical band in the 3200–3500 cm^−1^ region corresponds to the O-H and N-H stretching. The band confirmed the presence of a residual at around 1650 cm^−1^ (C=O stretching of Amide I) which represents the residual N-acetyl groups, and the absorption band at 1150 cm^−1^ attributed to the asymmetric stretching of the C-O-C bridge [42]. The acidified CS infrared spectrogram shows that the absorption band of amide I shifted to a lower frequency, and the O-H intensity was reduced. This phenomenon can be explained as the -NH_2_ protonation influenced the hydrogen bond among chitosan molecules. For the RHC molecule, a typical collagen band was found at 1625 cm^−1^, corresponding to the C=O stretching vibrations of Amide I. Amide II was found at 1545 cm^−1^, corresponding to N-H deformation vibrations. Amide A was found at 3290 cm^−1^, corresponding to N-H and O-H vibrations. υC-H corresponding to amide B was found at 3077 cm^−1^. The band at 1046 cm^−1^ was attributed to the C=C-H in-plane bending vibrations of RHCMA, indicating that methacryloyl substitutions occurred on the RHC molecules. Finally, the intensity of asymmetric stretching varied with CS to RHCMA ratios (Figure 2B).

### 3.2. Internal Morphology

**Figure 3:**
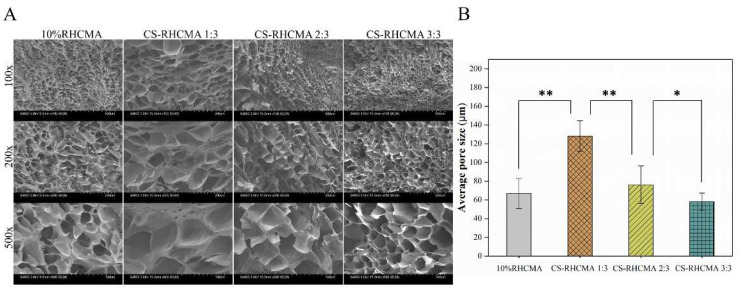
Internal morphology characterizations of the samples prepared from UV-cured bioinks. (**A**) SEM images of the internal morphology of the freeze-dried RHCMA and CS-RHCMA samples. Scale bar = 200 µm. (**B**) The summarized average pore size of the freeze-dried RHCMA and CS-RHCMA samples. Significance is indicated with * *p* < 0.05 and ** *p* < 0.01.

The internal morphologies of UV-cured RHC and CS-RHCMA samples are shown in Figure 3A. All samples share interconnected porous morphology with homogenous pore distributions. The addition of chitosan to the RHCMA solution increased the pore size, with an average pore size of 128 μm for CS-RHCMA 1:3 compared to 66 μm for 10% RHCMA. Further increasing the CS content in the bioinks leads to decreasing pore size and denser pore walls, as the CS-RHCMA 3:3 has the smallest pore size with 58 μm. A possible explanation for this phenomenon is that increasing the content of chitosan leads to an increase in the viscosity of the CS-RHCMA, and a mixture with a higher viscosity prevents crystallization when freeze-drying. A previous study also confirmed that a greater concentration of chitosan resulted in a smaller pore size in the prepared collagen-chitosan scaffolds [43]. As a result, the fabricated constructs’ internal pore size can be manipulated by the CS content, and manipulating the internal structures of bioinks can benefit the cell fate post-bioprinting since native tissue and organs usually have varied internal microenvironments.

### 3.3. Mechanical Properties and Biodegradation

**Figure 4:**
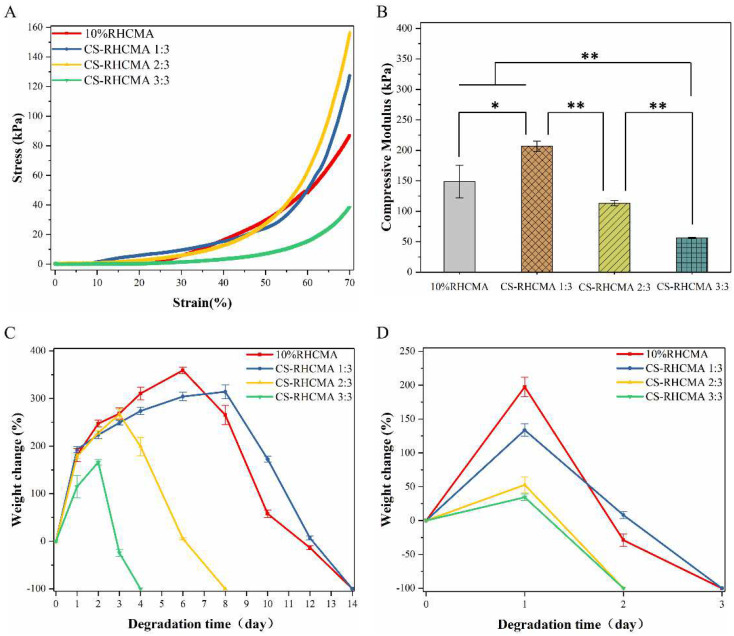
Characterizations of the samples prepared from UV-cured bioinks. (**A**) Representative stress-strain curves of RHCMA and CS-RHCMA samples. (**B**) Compressive modulus determined from RHC and CS-RHCMA samples, significance is indicated with * *p* < 0.05 and ** *p* < 0.01. (**C**) Degradation of RHCMA and CS-RHCMA samples incubated in PBS solution (pH 7.2). (**D**) Degradation of RHCMA and CS-RHCMA samples incubated in lysozyme solution (pH 6.5).

Bioinks formed constructs should have adequate mechanical strengths to maintain structural integrities and support cellular activities. Importantly, bioinks formed constructs should have tunable mechanical properties that fulfill the different applications since the mechanical properties of native human tissue differ from each other. This study conducted a compression test to illustrate the mechanical strengths of the UV-cured bioinks, and the results are shown in Figure 4. The representative stress-strain curves determined from the compression test indicated that the developed bioinks formed constructs performed with viscoelastic behaviors similar to some human soft tissues (Figure 4A), and the mechanical strengths comparable to the native tissues, such as skin and muscles [44,45].

Interestingly, adding acidified CS into RHCMA primarily increased the compressive modulus, while further increasing the CS ratio in CS-RHCMA reduced the compressive modulus (Figure 4B). As a result, the CS-RHCMA 1:3 sample has the highest mechanical strength (206.68 kPa), while the modulus of CS-RHC 3:3 was reduced to 56.27 kPa. A possible explanation for this result is that soluble free CS chains would interrupt RHCMA gelation during UV-induced polymerization. A similar phenomenon has been recorded in previous studies, where soft hydrogel systems composed of RHC and chitosan were investigated [46].

The RHCMA and CS-RHCMA samples’ degradation was tested in PBS or lysozyme solution, and the results are shown in Figure 4C,D. Samples incubated in lysozyme solution were completely degraded within 4 days versus 14 days in PBS solution. Moreover, RHC and CS-RHCMA 1:3 showed a slower degradation speed by further increasing the CS ratio in mixed bioinks resulting in faster degradation. These findings might be attributed to the mechanical properties of the UV-cured bioinks since adding extra acidified CS to CS-RHCMA decreased the mechanical strength by interrupting the gelation networks. As a result, UV-cured bioinks with higher mechanical strengths could resist the fast breakdown of their internal structures.

### 3.4. Viscosity and Printability

**Figure 5:**
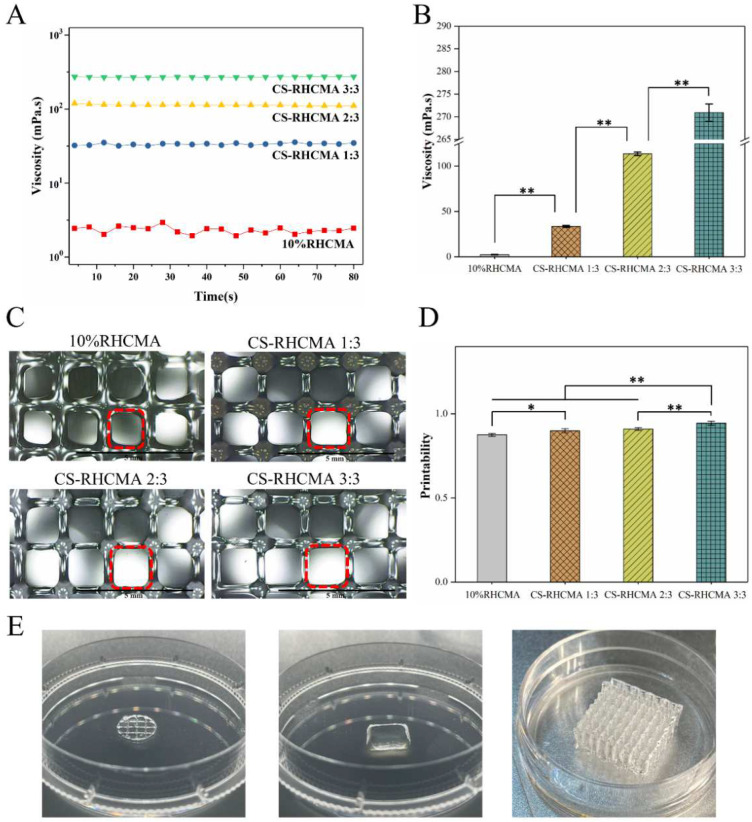
Viscosity and printing feasibility of RHCMA and CS-RHCMA bioinks. (**A**) Viscosity behaviors of the bioinks determined from rheological test. (**B**) Average viscosity value of the tested samples. (**C**) Optical microscope images of the printed lattices. Scale bar = 5 mm. (**D**) Determination of the printability of different bioinks. Significance is indicated with * *p* < 0.5 and ** *p* < 0.01. (**E**) 3D printed constructs different structures.

To understand the rheological property of the bioinks, the viscosity values of the bioinks were determined. Figure 5A,B summarize the viscosity property of the tested samples, and it shows adding CS to the 10% RHCMA increased its viscosity value. The viscosity shows an increasing trend as the content of CS increased in the CS-RHCMA bioinks. To assess the printability of the CS-RHCMA bioinks, lattice constructs were printed by extrusion-based 3D printing and UV-cured. Figure 5A shows that smooth filaments formed all printed constructs. The estimated printability of the bioinks was between 0.87 to 0.94 (Figure 4B). According to the criteria raised from a previous study, a printability value close to 1 indicates a well-performed 3D printing with sound structural fidelity and mechanical stability of the selected bioinks [41]. As the value of printability was significantly improved (*p* < 0.01) by introducing the acidified CS to the RHCMA bioinks, it confirmed our CS-RHCMA bioinks’ desired printability. Viscosity is one of the key parameters to the printability in extrusion-based bioprinting [47], and higher viscosity could lead to a precise printing result compared to the bioinks with relatively low viscosity [48]. Our results demonstrated the viscosity values of the bioinks were significantly enhanced by adding CS, and as a result, the printability was improved. Similar to our finding, a previous study emphasized chitosan, and chitosan–collagen gels were considered accurately printed bioinks because the relatively high viscosity of the solutions inhibited them from spreading out on the surface [49]. Constructs with different morphologies were 3D printed, and their images are shown in Figure 4C. These 3D printed constructs suggested that the CS-RHCMA bioinks can build complex structures for potential tissue engineering applications.

### 3.5. Cytotoxicity Test

**Figure 6:**
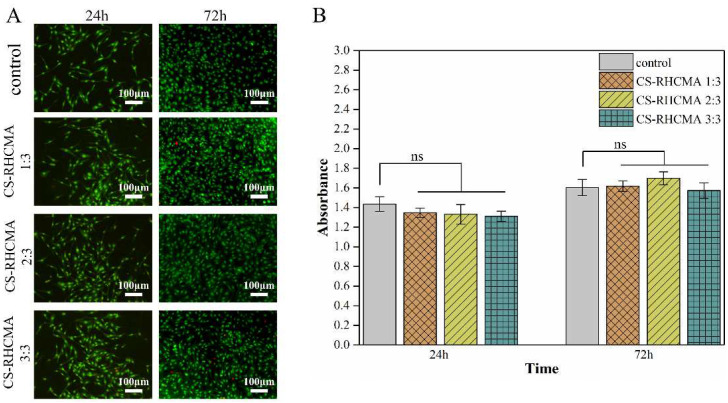
Cytotoxicity test of the CS-RHCMA bioinks. (**A**) Cellular morphology from the elution test was analyzed by AO/EB double-fluorescent staining after 24 and 72 h (living cells showing bright green, dead cells showing red). Scale bar = 100 µm. (**B**) Viability of HUVECs cultured in sample extracts assessed by CCK-8 assay at 24 and 72 h. None significant (ns) indicates *p* > 0.05.

To determine the cytotoxicity of the CS-RHCMA bioinks, cell morphology and survivability were evaluated by AO/EB double-fluorescent staining. As shown in Figure 6A, HUVECs exhibited green oval or spindle-like shapes in all groups after incubating in sample extracts for 24 h and 72 h, while dead cells in red color were seldom observed. The CCK-8 assay assessed the viability of HUVECs cultured in the sample extracts, where results were evaluated according to ISO-10993:5. Seeded HUVECs exhibited similar proliferation rates with no significant difference (*p* > 0.5) compared to the control group at the selected time points (Figure 6B). Furthermore, proliferation determined by CCK-8 at 72 h of culture was much higher than those cultured for 24 h, suggesting that extracts did not prevent cell proliferation. Altogether, the cytotoxicity tests demonstrated that all the UV-cured CS-RHCMA bioinks were cytocompatible and suitable for 3D bioprinting in vitro.

### 3.6. 3D Bioprinting HUVECs-Laden CS-RHCMA Bioinks

**Figure 7:**
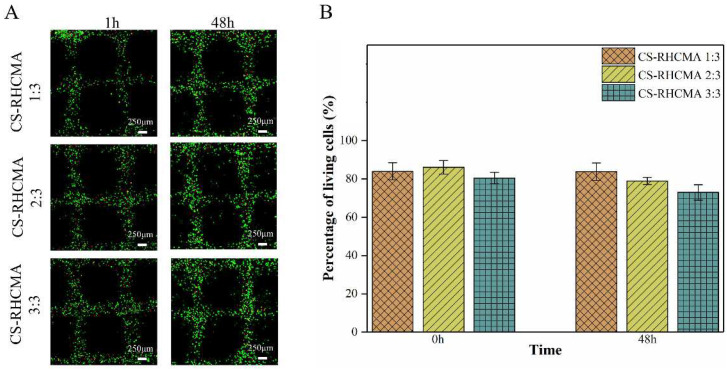
In vitro biocompatibility assessment of printed HUVECs-laden CS-RHCMA bioinks. (**A**) AO/EB double-fluorescent staining of the HUVECs in printed lattices at 1 and 48 h post-printing, respectively. Scale bar = 250 µm. (**B**) Calculated percentage of living cells within the bioprinted lattices.

The CS-RHCMA bioinks were adopted to load HUVECs for bioprinting, then the printed constructs were UV-cured for 45 s immediately. The HUVECs within the printed structures were visualized by AO/EB double-fluorescent staining, as shown in Figure 7A. The printed HUVECs were well sustained within the lattices prepared from three CS-RHCMA samples as nearly 80% of the cells were alive after the extrusion-based printing. In addition, there was no significant difference in the viabilities between the two time points post-printing (Figure 7B). Our results are comparable to previous studies where HUVECs were 3D bioprinted through other biomaterials such as alginate and GeLMA [50]. Thus, the CS-RHCMA bioinks system developed in the current study demonstrated reliable 3D bioprinting activities.

## 4. Conclusions

This research added the acidified chitosan to RHCMA solution to prepare photo-responsive bioinks for 3D printing. The mechanical strengths and internal pore size of the CS-RHCMA bioinks formed constructs that were tunable by varying the CS to RHCMA ratio. The biodegradation ability of these bioinks formed constructs was also influenced. Introducing CS to RHCMA improved the printability of the bioinks as 3D constructs were well-built through extrusion-based 3D printing. More importantly, these CS-RHCMA bioinks demonstrated great biocompatibility that supports the viability of the HUVECs within the printed constructs. In conclusion, the developed CS-RHCMA bioinks in the current study showed their potential in 3D bioprinting and tissue engineering. It is hoped that a complicated tissue structure with biological activities will be constructed in the future.

## Data Availability

Not applicable.
